# C6 Peptide-Based Multiplex Phosphorescence Analysis (PHOSPHAN) for Serologic Confirmation of Lyme Borreliosis

**DOI:** 10.1371/journal.pone.0130048

**Published:** 2015-07-06

**Authors:** Vera G. Pomelova, Edward I. Korenberg, Tatiana I. Kuznetsova, Tatiana A. Bychenkova, Natalya I. Bekman, Nikolay S. Osin

**Affiliations:** 1 Laboratory of Molecular Diagnostics, Department of Biological Microassay, State Research Institute of Biological Engineering, Moscow, Russian Federation; 2 Department of Infections with Natural Focality, N. F. Gamaleya Research Institute of Epidemiology and Microbiology, Moscow, Russian Federation; 3 Laboratory of Clinical Immunology, Clinical Infectious Diseases Hospital, Perm, Russian Federation; 4 Immunoscreen Closed Joint Stock Company, Moscow, Russian Federation; University of Kentucky College of Medicine, UNITED STATES

## Abstract

**Background:**

A single-tier immunoassay using the C6 peptide of VlsE (C6) from *Borrelia burgdorferi* sensu stricto (*Bb)* has been proposed as a potential alternative to conventional two-tier testing for the serologic diagnosis of Lyme disease in the United States and Europe.

**Objective:**

To evaluate the performance of C6 peptide based multiplex Phosphorescence Analysis (PHOSPHAN) for the serologic confirmation of Lyme borreliosis (LB) in Russian patients.

**Methods:**

Serum samples (*n* = 351) were collected from 146 patients with erythema migrans (EM); samples from 131 of these patients were taken several times prior to treatment and at different stages of recovery. The control group consisted of 197 healthy blood donors and 31 patients with other diseases, all from the same highly endemic region of Russia. All samples were analyzed by PHOSPHAN for IgM and IgG to *Bb* C6, recombinant OspC and VlsE proteins, and C6 peptides from *B*. *garinii* and *B*. *afzelii*.

**Results:**

IgM and IgG to *Bb* C6 were identified in 43 and 95 out of 131 patients (32.8 and 72.5%, respectively); seroconversion of IgM antibodies was observed in about half of the patients (51.2%), and of IgG antibodies, in almost all of them (88.4%). Additional detection of OspC-IgM and VlsE-IgM or IgG to C6 from *B*. *garinii* or *B*. *afzelii* did not contribute significantly to the overall sensitivity of the multiplex immunoassay.

**Conclusions:**

The multiplex phosphorescence immunoassay is a promising method for simultaneously revealing the spectrum of antibodies to several *Borrelia* antigens. Detection of IgM and IgG to *Bb* C6 in the sera of EM patients provides effective serologic confirmation of LB and, with high probability, indicates an active infection process.

## Introduction

Ixodid Tick-borne Borrelioses, infections of the Lyme Borreliosis (LB) group in Russia [1] (hereinafter referred to as LB), are classic transmissible infections. They are caused by spirochetes of the *Borrelia burgdorferi* sensu lato group transmitted by ixodid ticks. Among human-pathogenic *Borrelia*, the most widespread are *B*. *burgdorferi* sensu stricto (*Bb*), which causes Lyme disease in North America and Europe, and also Eurasian *B*. *garinii* (*Bg*) and *B*. *afzelii* (*Ba)* [2, 3]. The last two genospecies are etiologic agents of almost all LB cases in Russia, which encompasses the greater part of the range of *B*. *burgdorferi* sensu lato [3]. Cases of LB are being recorded in almost 70 administrative regions of the Russian Federation, from the Baltic region to Southern Sakhalin. According to official statistics, the annual number of confirmed LB cases in 2012 reached approximately 8300, which was equivalent to 5.8 cases per 100 000 population.

LB disease affects the skin, connective tissue, and nervous and cardiovascular systems, with its clinical manifestations in the United States significantly differing from those in Europe [2, 4, 5, 6]. Typical erythema migrans (EM) is the only pathognomonic sign of early LB.

Serologic methods are regarded as basic for laboratory confirmation of LB at all stages after the first few weeks of disease progression [7–9]. The conventional two-tier procedure of testing for *Borrelia* infection in the United States and Europe is based on a combination of enzyme-linked immunosorbent assay (ELISA) or, less frequently, indirect immunofluorescence analysis with immunoblotting [7, 8, 10]. A two-tier testing with *Bb* whole-cell sonicate is not used in Russia because of well known disadvantages of the immunoblot method and the lack of standardized interpretation criteria for immunoblot bands, which is particularly a problem in regions where multiple genostecies of *Borrelia* co-circulate. Recent progress in methods for LB serologic diagnosis is largely due to the development of tests based on the immunodominant C6 peptide that has the same amino acid sequence as the conserved region (IR6) of *B*. *burgdorferi* VlsE surface protein [11], and also to advances in detection of anti-*Borrelia* IgG and IgM in ELISAs based on combinations of VlsE and OspC proteins [3] or their peptide fragments [12, 13], including a multiplex variant of the assay [14]. In Europe, it is recommended to use *Bb* C6 peptide in combination with peptides reproducing the amino acid sequences of the immunodominant IR6 domains of European *Borrelia* genospecies [15–18] and with recombinant proteins, primarily OspC and VlsE [19, 20]. Multiplex multiantigen tests, which permit evaluation of many antibodies in the same serum sample [14, 21], appear promising, particularly in cases of mixed infection [21].

This study deals with the results of using a multiplex immunoassay based on the microplate microarray phosphorescence analysis (PHOSPHAN) technology (Immunoscreen, Russia) [22] for the detection of immunoglobulins M and G to C6 peptides and of immunoglobulins M to OspC or VlsE proteins of *B*. *burgdorferi* sensu lato. IgG responses to OspC and VlsE were not included in the analysis since they had no significant contribution to the total C6-IgG responses measured in EM patients [21]. The purpose of this study was to evaluate the performance of C6 peptide based PHOSPHAN for the serologic confirmation of Lyme borreliosis (LB) in patients with erythema migrans in the acute period of the disease. The efficiency of using this approach was analyzed as a function of the incubation period (after tick bite), the duration of EM prior to treatment, and the duration of the infection process (time after initiation of antibiotic therapy). The frequency of antibody detection and seroconversion in tests of sequential serum samples was determined.

## Materials and Methods

### Ethics Statement

The study was approved by the Institutional Review Board (Scientific Technical Council) of the State Research Institute of Biological Engineering, Moscow (protocol number 2010/3135/11 and 2014/06/16). Patient informed consent was not required since the case histories of the patients and samples of their sera were anonymized and de-identified prior to analysis to protect subject confidentiality.

### Serum samples

The anonymized patient records and de-identified serum samples were provided in kind by Clinical Infectious Diseases Hospital, Perm, Russian Federation.

#### Sera from LB Patients

According to patient records, serum samples (N = 351) were taken from 146 patients with EM in a highly endemic region of Russia (Perm Krai) during the epidemic season (May to September) of 2010. People in this region are attacked only by adult *Ixodes persulcatus* ticks, in which *B*. *garinii* and *B*. *afzelii* prevail [23]. All the patients noted an attached tick before the onset of the disease. The clinical diagnosis of LB was based on the presence of typical EM, which was sometimes combined with a general infection syndrome; in 43 patients (29.5%), the clinical diagnosis was confirmed by PCR detection of *B*. *burgdorferi* sensu lato DNA in blood. The age of the patients averaged 59.7 ± 14.8 years; the period between tick bite and the onset of the disease varied from 1 to 47 days, the median being 9 days. All the patients were treated with doxycycline or less frequently with azithromycin at first visit to a clinic, shortly after the first blood sample was drawn [5]. Serum samples from 131 patients (89.7%) were taken two to five times, at different stages of recovery. Duration of EM prior to treatment was less than 7 days in 94 patients (64.4%), 7 to 14 days in 36 patients (24.7%), more than 14 days in 16 patients (11%). Serum samples from 15 individuals (10.3%) were taken only once at first visit to a clinic. Serum samples from 131 patients (89.7%) were taken two to four times, the first sample being taken at the baseline and the other samples taken at different stages of recovery. Two, three or four serum samples were taken from each of 60 patients (41.1%), 68 patients (46.6%) or three patients (2.1%), respectively. All serum samples were tested separately.

#### Control Groups

The control group (free of current *Borrelia* infection) included 197 healthy blood donors from the same endemic region and 31 patients with syphilis, leptospirosis, Epstein–Barr virus infection, or other diseases that could have an effect on the specificity of antibody detection in serologic tests for LB. Serum samples were divided into aliquots and stored before analysis at –20°C.

#### Control Sera

To control the quality of measurements in the PHOSPHAN assay, "positive" and "negative" control sera were prepared by pooling several sera that either contained specific antibodies to *B*. *burgdorferi* sensu lato according to C6 ELISA or contained no such antibodies.

### C6 ELISA

All serum samples were tested for the presence of total IgM/IgG to *B*. *garinii* strain Ip90 using the Immunetics C6 Lyme ELISA Kit (Immunetics Inc., USA) according to the manufacturer's instructions.

### Peptide and Recombinant Antigens

The C6 peptides from *Bb* B31 and *Bg* Ip90 were kindly provided by Dr. Barbara Johnson (CDC, Fort Collins, CO). The C6 peptide of *Ba* ACA-1 was synthesized by Verta (St. Petersburg, Russia). The structure of these peptides reproduced that described by Sillanpaa et al. [16]. To allow efficient adsorption in microplates, the peptides were conjugated with BSA (Sigma, United States) [24]. Recombinant OspC and VlsE proteins were from Omnix (St. Petersburg). Each of them was a chimeric recombinant antigen of three *Borrelia* genospecies (*Bb* B31, *Bg* Ip90, and *Ba* ACA-1).

### PHOSPHAN Multiplex Immunoassay

PHOSPHAN is a solid-phase sandwich immunoassay performed in standard Maxisorp 96-well microplates (Nunc, Denmark), similar to ELISA [21, 22] (S1 Fig). A nanoplotter system for contact printing (Immunoscreen) was used to print an array of nine dots (0.7 mm in diameter) on the well bottoms. The dots contained combinations of C6 *Bb* B31 and recombinant OspC and VlsE (for IgM detection) or C6 *Bb* B31 and C6 from *Bg* Ip90 and *Ba* ACA-1 (for IgG detection). The samples were diluted 100-fold in reaction buffer (12.1 mg/mL Tris-HCl, pH 7.75, NaCl 8.7 mg/ml, NaN_3_ 0.5 mg/mL, BSA 5 mg/mL, 0.01% Tween 20), 200- μL aliquots were loaded in the wells, and the microplate was incubated on a shaker at room temperature for 2 h. Thereafter, the wells were treated with monoclonal mouse antibodies to human IgM or IgG (Sorbent-Service, Moscow, Russia) conjugated with biotin (500 ng/mL) and then with streptavidin conjugated with platinum coproporphyrin (300 ng/mL) (100 and 30 μL per well, incubation time 60 and 15 min, respectively). Each stage was followed by washing in three portions of wash buffer (8.7 g/L NaCl, 0.5 mg/mL Tween 20, 0.2 mg/mL NaN_3_); at the final stage, the microplate was additionally washed in three portions of distilled water and dried in the air. Every sample was analyzed in duplicate (in two wells). The positive and negative control sera were included in each experiment. Phosphorescence intensity was measured by scanning the microplates in a Diagem biochip analyzer (Immunoscreen) in a time-resolved mode (S2 Fig). The results were expressed as total number of photoelectric pulses generated from each dot. After computer processing, the pattern of phosphorescence signal distribution over the well bottom was represented by nine colored spots (three per antigen), with color intensity proportional to the concentration of IgM or IgG in the sample (S3 Fig). On this basis, values of the Lyme Index (*LI*) were calculated: *LI = P /* (*N* + *b*), where *P* and *N* are phosphorescence intensities of the sample and the negative control serum, respectively, for each of the six antigens. In optimization experiments, the values of *b* were selected so as to ensure at least 95% specificity when 100 donor sera were tested. These values for IgG with C6 from *Bb*, *Bg*, and *Ba* were 50, 40, and 40 pulses; for IgM with *Bb* C6 and OspC or VlsE, they were 400, 200, or 200 pulses, respectively. The average phosphorescence intensity for each antigen was calculated from six measurements (two wells with three active dots in each). The results were considered positive (IgM or IgG detected) at *LI* ≥ 1, on the condition that the phosphorescence intensity recorded with the positive control serum was at least 1000 pulses, and with the negative control serum, no more than 200 pulses.

### Statistical Analysis

Differences between proportions were considered significant at 2-tailed *P* ≤0.05 (Fisher's exact test).

## Results

### Performance of PHOSPHAN and C6 ELISA

**Fig 1:**
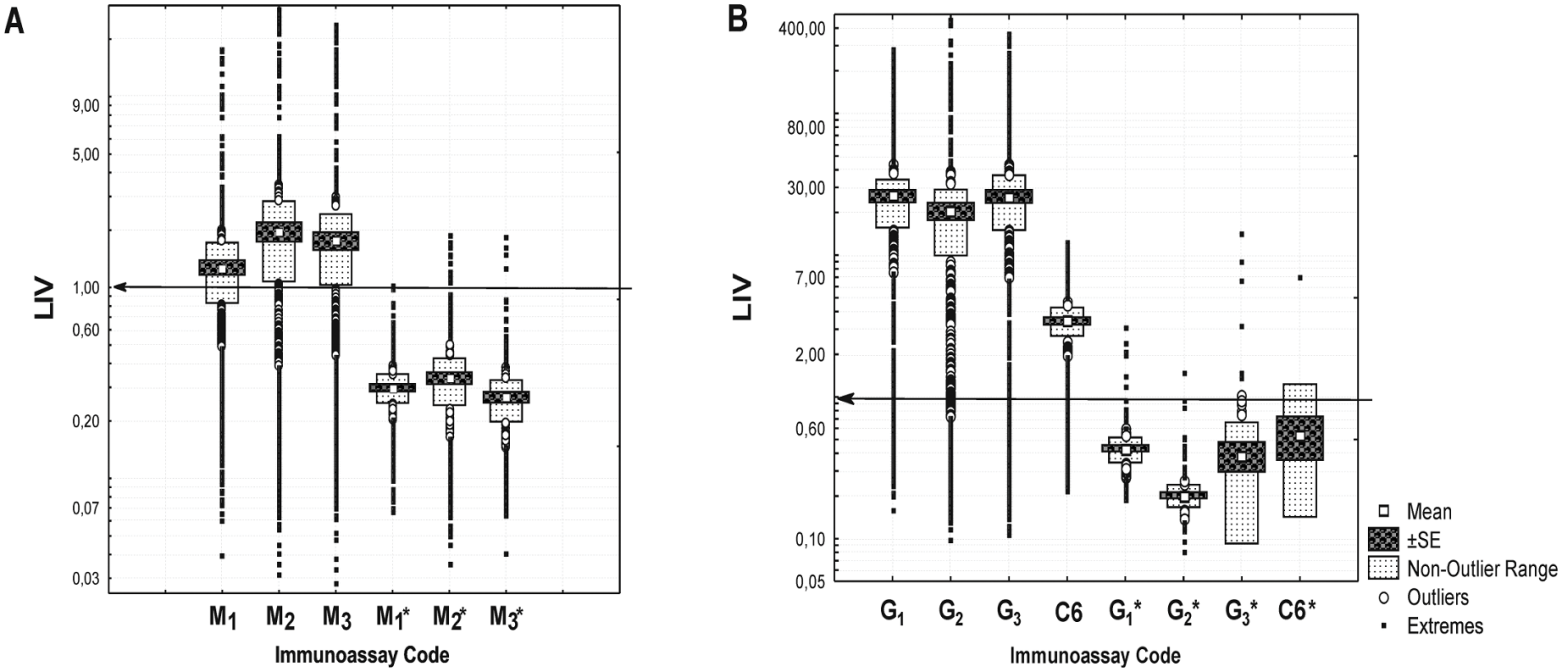
Lyme Index values (LIV) for sera from LB patients with erythema migrans (N = 351) and healthy blood donors (N = 197). (A) PHOSPHAN variants M_1_–M_3_ and (B) PHOSPHAN variants G_1_–G_3_ and C6 ELISA (C6). Blood donors, *; line with arrowhead, threshold level (LIV = 1). Immunoassay codes: M_1_, *Bb* C6 IgM; M_2_, OspC IgM; M_3_, VlsE IgM; G_1_, *Bb* C6 IgG; G_2_, *Bg* C6 IgG; G_3_, *Ba* C6 IgG; C6, Immunetics C6 Lyme ELISA kit. (*Bb*) *B*.*burgdorferi*, (*Bg*) *B*.*garinii*, (*Ba*) *B*.*afzelii*. Legend: Transparent square, Mean; Spotted rectangle, ± Standard Error; Dotted rectangle, Non-Outlier Range; Transparent circle, Outliers; Black square, Extremes.

Fig 1 illustrates the results of IgM and IgG detection with each of six antigens by PHOSPHAN and of IgM/IgG detection by C6 ELISA. Both methods allowed differentiation between LB patients and healthy donors. In PHOSPHAN tests, the average *LI* values for IgM detection with antigens *Bb* C6 (M_1_), OspC (M_2_), or VlsE (M_3_) in the healthy donors were in the range of 0.27–0.34, compared to 1.28–1.97 in the LB group (Fig 1A). The respective values for IgG detection with antigens *Bb* C6 (G_1_), *Bg* C6 (G_2_), or *Ba* C6 (G_3_) were 0.17–0.37 and 20.6–26.2; in C6 ELISA, they were 0.54 (95% CI: 0.12, 2.1) and 3.4 (95% CI: 0.2, 7.2) (Fig 1B). Compared to the control samples, the average *LI* values for the LB group were higher by factors of 5.6 (IgM detection) and 70 (IgG detection) in PHOSPHAN tests and by a factor of 6.4 in the C6 ELISA. The highest *LI* values for serum samples from the LB group ranged from 18–29 in PHOSPHAN variants M_1_, M_2_, or M_3_ (Fig 1A) and from 287–462 in variants G_1_, G_2_, or G_3_; the highest values recorded in the C6 ELISA did not exceed 12.3 (Fig 1B). Coefficients of variation in the *LI* values obtained after repeated analysis of the same samples on different days were slightly higher in PHOSPHAN than in the C6 ELISA (up to 25% vs. no more than 15%).

### Specificity of PHOSPHAN and C6 ELISA

In PHOSPHAN tests of serum samples from donors, the specificity of IgM detection with antigens C6 (M_1_), OspC (M_2_) and VlsE (M_3_) or of IgG detection with *Bb* C6 (G_1_), *Bg* C6 (G_2_) and *Ba* C6 (G_3_) reached 94.4–99.5%, which was comparable to the parameters recorded in C6 ELISA and PHOSPHAN variant (M_1_G_1_) for detecting total IgM + IgG to *Bb* C6 (Table 1). A somewhat lower specificity was observed in tests for any IgM or IgG (M_1–3_, or G_1–3_) with the lowest specificity recorded in PHOSPHAN variant M_1–3_/G_1–3_. The specificity of both methods decreased when samples from patients with diseases other than LB were included in analysis. In PHOSPHAN tests, this decrease was especially evident in cases of IgM detection with VlsE (M_3_) or with any of the three (M_1–3_) or six antigens (M_1–3_/G_1–3_). Because of the significantly lower specificity of PHOSPHAN variant M_1–3_/G_1–3_ it was excluded from further analysis.

**Table 1:** Specificity of PHOSPHAN and C6 ELISA Tests.

Assay code	Antigen	Detected antibodies	Number (%) [95% CI] of negative samples^a^	
			Healthy donors (N = 197)	Patients with non-Borrelia infections (N = 31)
M_1_	*Bb* C6	IgM	195 (99.0) [96.4, 99.9]	31 (100)
M_2_	OspC	IgM	186 (94.4) [90.2, 97.2]	29 (93.5) [78.5, 99.2]
M_3_	VlsE	IgM	193 (98.0) [94.9, 99.5]	22 (71) [52.0, 85.8]
M_1–3_	Any of the three	IgM	181 (91.9) [87.1, 95.3]	21 (67.7) [48.6, 83.3]
G_1_	*Bb* C6	IgG	191 (97) [93.5, 98.9]	29 (93.5) [78.5, 99.2]
G_2_	*Bg* C6	IgG	196 (99.5) [97.2, 99.9]	30 (96.8) [83.3, 99.9]
G_3_	*Ba* C6	IgG	190 (96.4) [92.7, 98.5]	31 (100)
G_1–3_	Any of the three	IgG	182 (92.4) [87.7, 95.7]	29 (93.5) [78.5, 99.2]
M_1–3_/G_1–3_	Any of the six	IgM, IgG	166 (84.3) [78.4, 89.1]	21 (67.7) [48.6, 83.3]
M_1_G_1_	*Bb* C6	IgM, IgG	189 (95.9) [92.1, 98.2]	29 (93.5) [78.5, 99.2]
C6	C6 ELISA^b^	IgM, IgG	96 (96)^c^ [90.1, 98.9]	186 (91.6)^d^ [86.9, 95.0]

*(Bb) B. burgdorferi, (Bg) B. garinii, (Ba) B. afzelii.*

^a^Statistically significant differences (Fisher's exact test, *p* < 0.05) were observed for comparison of pairs: M_1_ versus M_1–3_, G_2_ versus G_1–3_, M_1–3_/G_1–3_ versus G_1_, G_2_ and G_3_ or M_1_, M_2_ and M_3_ in the donor group, or M_3_ versus M_3_ and M_1–3_ versus M_1–3_ in the groups of donors and patients with non-*Borrelia* infections. All other comparisons were not statistically significant.

^b^Immunetics C6 Lyme ELISA Kit.

^c^N = 100.

^d^N = 203 (these included 31 samples tested in PHOSPHAN + 172 from the same set of sera described previously [21]).

### Sensitivity of *Bb* C6 peptide based PHOSPHAN and C6 ELISA

At the baseline, *Bb* C6 peptide based PHOSPHAN and C6 ELISA positivity correlated directly with EM duration except for *Bb* C6 IgM test. Number of positive samples detected by both methods was significantly greater in patients with EM duration of ≥7 days than in patients with EM duration of <7 days. Positive PHOSPHAN reaction with C6 from *B*. *burgdorferi* was observed more frequently in tests for IgG than for IgM; however these differences reached statistically significant levels only in patients with EM duration of more than 14 days. Sensitivity of the PHOSPHAN variant detecting total IgM+IgG was significantly higher as compared to C6 ELISA only in patients with EM duration of <7 days (Table 2).

**Table 2:** Sensitivity of *Bb* C6 peptide based PHOSPHAN and C6 ELISA tests for serum IgM and IgG antibody detection in samples from EM patients prior to treatment (N = 146).

Assay variant	Number (%) [95% CI] of positive serum samples at the baseline in patients with EM as a function of rash duration^a^		
	<7 days (N = 94)	7–14 days (N = 36)	>14 days (N = 16)
*Bb* C6 IgM	21 (22.3) [14.4, 32.1]	10 (27.8) [14.1, 45.2]	3 (18.8) [4.1, 45.7]
*Bb* C6 IgG	26 (27.7) [19.0, 37.9]	21 (58.3) [40.7, 74.5]	12 (75) [47.6, 92.7]
*Bb* C6 IgM+IgG	37 (39.4) [29.5, 50]	25 (69.4) [51.8, 83.6]	12 (75) [47.6, 92.7]
C6 ELISA^b^	16/85^c^ (18.8) [11.5, 28.2]	18/32 (56.3) [38.9, 72.7]	8/10 (80) [44.4, 97.5]

*(Bb) B. burgdorferi, (Bg) B. garinii, (Ba) B. afzelii.*

^a^Statistically significant differences (Fisher's exact test, *p* < 0.05) were observed for comparison of pairs: C6 ELISA versus *Bb* C6 IgM+IgG at EM duration <7 days; *Bb* C6 IgM versus *Bb* C6 IgM+IgG at EM duration 7–14 days; *Bb* C6 IgM versus *Bb* C6 IgG and *Bb* C6 IgM+IgG at EM duration >14 days; <7 days versus 7 to 14 days for *Bb* C6 IgG, *Bb* C6 IgM+IgG and C6 ELISA; <7 days versus >14 days for *Bb* C6 IgG and C6 ELISA. All other comparisons were not statistically significant.

^b^Immunetics C6 Lyme ELISA Kit.

^c^Number of positive serum specimens/total number of serum specimens tested.

**Fig 2:**
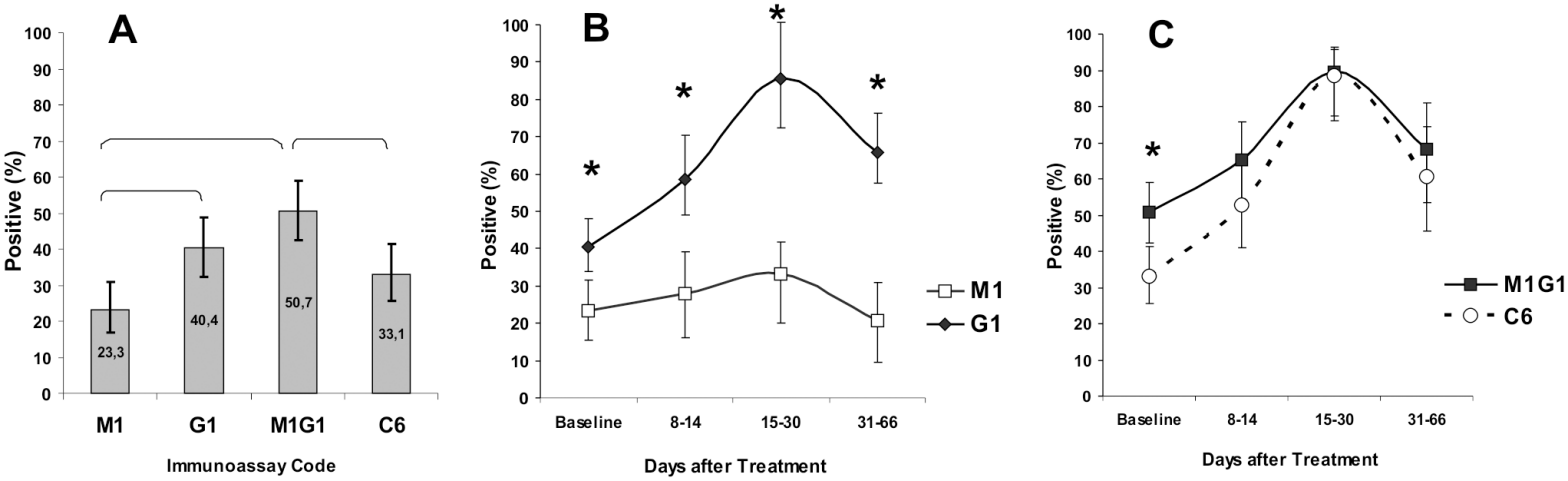
Sensitivity of PHOSPHAN and C6 ELISA tests for serum IgM and IgG antibody responses to *B*. *burgdorferi* C6 in samples from EM patients. (A) Sensitivity of PHOSPHAN variants M_1_, G_1_, M_1_G_1_ and C6 ELISA (C6) at the baseline prior to treatment (N = 146). (B) Sensitivity of PHOSPHAN variants M_1_ and G_1_, and (C) PHOSPHAN variant M_1_G_1_ and C6 ELISA in tests of serum samples taken at the baseline (n = 146) and on days 8–14 (*n* = 75), 15–30 (n = 48), and 31–66 (*n* = 82) after disease onset. Values that are statistically significant (Fisher's exact test, *p* < 0.05) are denoted by brackets or asterisks. Immunoassay codes: M_1_, *Bb* C6 IgM; G_1_, *Bb* C6 IgG; M_1_G_1_, *Bb* C6 IgM+IgG; C6, Immunetics C6 Lyme ELISA kit. (*Bb*) *B*.*burgdorferi*. Legend: (B) Transparent square, M1; Black diamond, G1. (C) Black square, M1G1; Transparent circle, C6.

In general, a positive PHOSPHAN reaction with C6 from *B*. *burgdorferi* was observed significantly more frequently in tests for IgG (G_1_) than for IgM (M_1_) both at the baseline prior to treatment and at all time intervals postbaseline (Fig 2A and 2B). The frequency of positive samples in tests for both IgM and IgG (M_1_G_1_) was higher than in variant G_1_ (Fig 2A), although the difference lacked statistical significance (*p* = 0.07). The sensitivity of C6 ELISA was comparable to that of the PHOSPHAN variant for IgG detection (G_1_), but it was significantly lower than the total IgM and IgG variant assay (M_1_G_1_) (Fig 2A). Difference in sensitivity between PHOSPHAN and C6 ELISA was especially apparent at the baseline (*p* < 0.05), whereas no statistically significant difference was observed at the later time intervals after start of treatment (Fig 2C).

### Detection of IgM to OspC and VlsE in PHOSPHAN

At the baseline, a positive PHOSPHAN reaction for IgM detection with OspC was observed in 13.8% (95% CI: 7.6, 22.5) of patients with EM of <7 days of duration, whereas it was observed in 27.8% (95% CI: 14.1, 45.2) and 31.3% (95% CI: 11.1, 58.7) of patients with EM of 7 to 14 days and >14 days in duration, respectively. The respective values for IgM detection with VlsE were 4.3% (95% CI: 1.2, 10.6), 25% (95% CI: 12.1, 42.2) and 37.5% (95% CI: 15.2, 64.6). A significantly lower sensitivity (p<0.05) was observed only for IgM detection with VlsE versus *Bb* C6 in patients with EM of <7 days duration.

**Fig 3:**
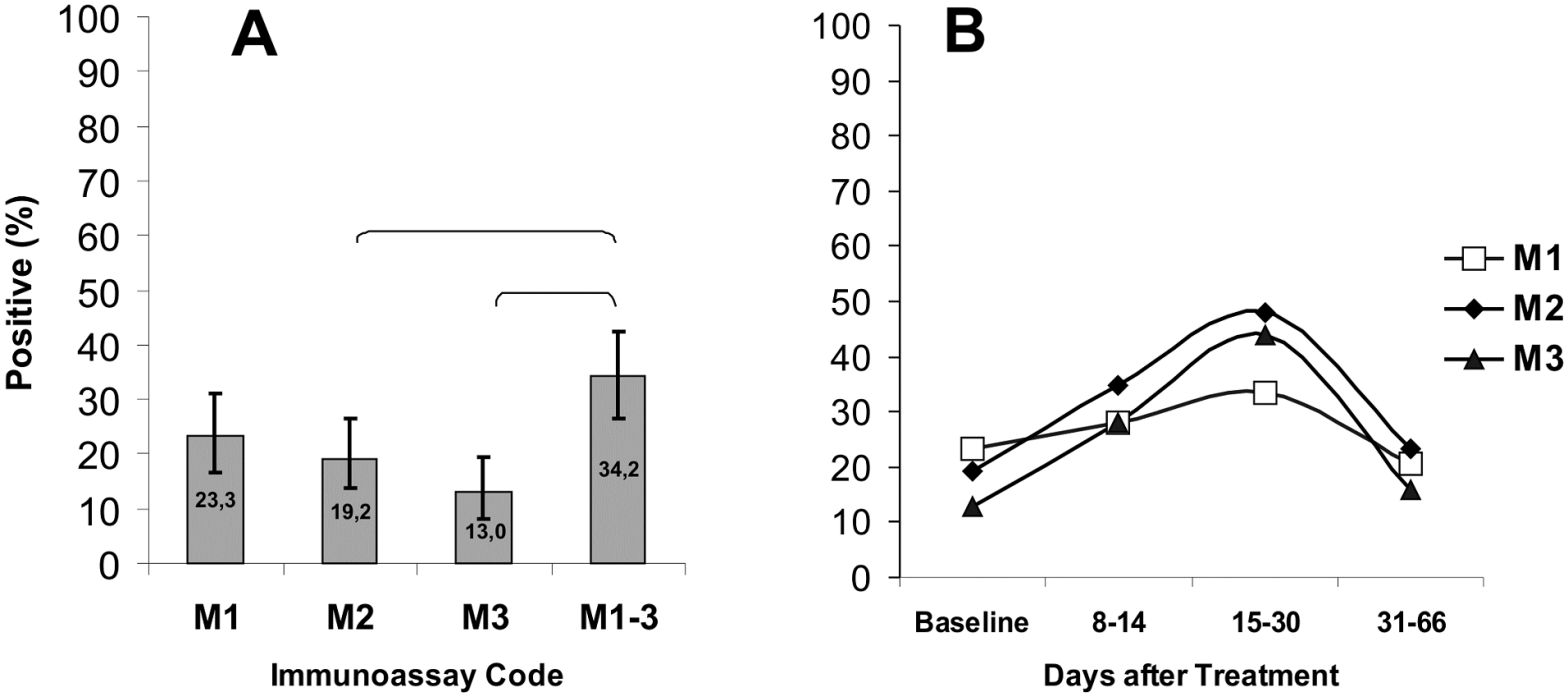
Sensitivity of PHOSPHAN tests for serum IgM antibody responses to *B*. *burgdorferi* C6, OspC, and VlsE in samples from EM patients. (A) Total sensitivity of PHOSPHAN variants M_1_, M_2_, M_3_ and M_1–3_ at the baseline prior to treatment (N = 146). (B) Sensitivity of PHOSPHAN variants M_1_–M_3_ in tests of samples taken at the baseline (n = 146) and on days 8–14 (*n* = 75), 15–30 (n = 48), and 31–66 (*n* = 82) after disease onset. The brackets indicate that the difference between M_1–3_ and M_3_ or M_1–3_ and M_2_ is statistically significant (Fisher's exact test, *p* < 0.05). Immunoassay codes: M_1_, *Bb* C6 IgM; M_2_, OspC IgM; M_3_, VlsE IgM; M_1–3_, any of the three antigens *Bb* C6, OspC or VlsE. (*Bb*) *B*.*burgdorferi*. Legend: (B) Transparent square, M1; Black diamond, M2; Black triangle, M3.

In general, the IgMs reacting with antigens *Bb* C6 *(*M_1_), OspC (M_2_), or VlsE (M_3_) were detected in 13–23.3% serum samples from LB patients at the baseline prior to treatment. The frequency of IgM detection with any of the three antigens (M_1–3_) was significantly higher than with VlsE or OspC antigens (Fig 3A). At all time intervals postbaseline, differences in IgM detection frequency between individual antigens lacked statistical significance (Fig 3B). However, positive reactions with OspC or VlsE were observed only in the samples (both at the baseline and at different times of recovery) from patients that were also positive for IgM and/or IgG to *Bb* C6 (variant M_1_G_1_). Therefore, additional detection of IgM to recombinant proteins did not improve significantly the overall sensitivity of the latter PHOSPHAN variant.

### Detection of IgG to C6 from *B*. *garinii* or *B*. *afzelii* in PHOSPHAN

At the baseline, the frequency of positive PHOSPHAN reactions for IgG detection with *Bg* C6 or *Ba* C6 peptides did not differ significantly from the parameters observed for IgG detection with *Bb* C6 in patients with different duration of EM (Table 2). A significantly higher sensitivity (p<0.05) was observed for IgG detection with *Bg* C6 or *Ba* C6 peptides in patients with EM of >14 days duration versus <7 days duration.

**Fig 4:**
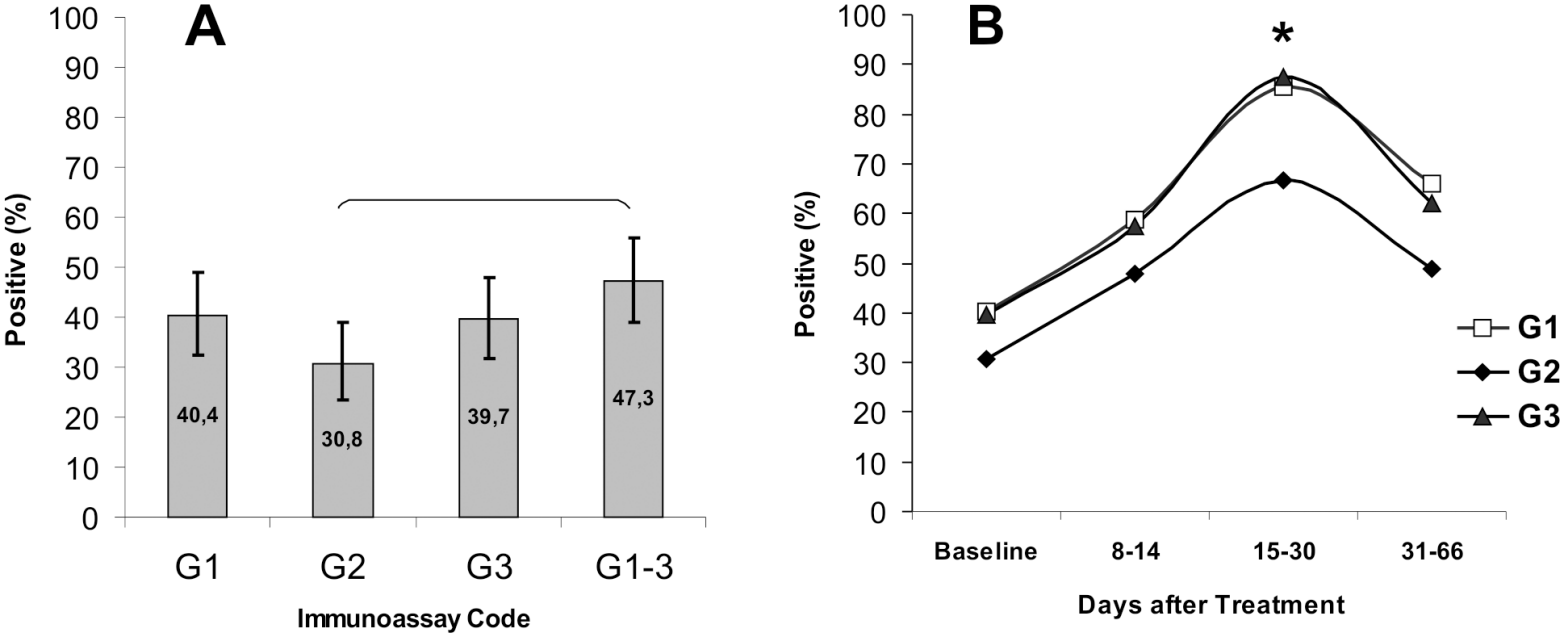
Sensitivity of PHOSPHAN tests for serum IgG antibody responses to *B*. *burgdorferi* C6, *B*. *garinii* C6, and *B*. *afzelii* C6 in samples from EM patients. (A) Total sensitivity of PHOSPHAN variants G_1_, G_2_, G_3_, and G_1–3_ at the baseline prior to treatment (N = 146). (B) Sensitivity of PHOSPHAN variants G_1_–G_3_ in tests of samples taken at the baseline (*n* = 146) and on days 8–14 (*n* = 75), 15–30 (n = 48), and 31–66 (*n* = 82) after disease onset. Values that are statistically significant (Fisher's exact test, *p* < 0.05) are denoted by brackets or asterisks. Immunoassay codes: G_1_, *Bb* C6 IgG; G_2_, *Bg* C6 IgG; G_3_, *Ba* C6 IgG; G_1–3_, any of the three antigens *Bb* C6, *Bg* C6, or *Ba* C6. (*Bb*) *B*.*burgdorferi*, (*Bg*) *B*.*garinii*, (*Ba*) *B*.*afzelii*. Legend: (B) Transparent square, G1; Black diamond, G2; Black triangle, G3.

In general, positive reactions for specific IgG with *Bb* C6 (G_1_), Bg C6 (G_2_), or *Ba* C6 (G_3_) were detected in 30.8–40.4% serum samples from LB patients at the baseline prior to treatment. The frequency of IgG detection with any of the three antigens (G_1–3_) was significantly higher only as compared to G_2_ (Fig 4A). In the course of the disease, the frequency of detection of IgG to *Bb* C6 increased from 40.4% at the baseline (95% CI: 32.4, 48.8) to a peak of 85.4% on days 15–30 after disease onset (95% CI: 72.2, 93.9) and then began to decrease. Parameters observed with *Ba* C6 were similar, while those with *Bg* C6 were markedly lower. Differences in IgG detection frequency between *Bg* C6 and two other C6 peptides were statistically significant only on days 15–30 from onset of illness (Fig 4B).

### The Frequency of Antibody Seroconversion in EM Patients

The frequency of seroconversion was evaluated in 131 EM patients, from each of which at least two serum samples were taken. The first serum sample was taken from a patient at the baseline prior to treatment, the second, third or fourth samples were taken from the same patient on 8–14 days or later times of recovery. In optimization experiments for the end-point titration of sequential serum samples, the values of the Lyme Index in PHOSPHAN correlated directly with the end-point titer. A 4-fold increase or decrease in antibody titer corresponded to no less than a two-fold increase or decrease in Lyme Index values with individual *Borrelia* antigens (data are not shown). With this in mind, seroconversion in the present study was ascertained by the appearance of IgM or IgG and/or by no less than a two-fold increase or decrease in Lyme Index values in sequential serum samples.

Of the total, positive PHOSPHAN reactions were observed in 95 patients (72.5%) for IgG to *Bb* C6 and *Ba* C6, in 77 (58.8%) for IgG to *Bg* C6, in 43 (32.8%) for IgM to *Bb* C6, in 52 (39.7%) for IgM to OspC, and in 45 (34.4%) for IgM to VlsE (Table 3).

**Table 3:** Seroconversion in EM patients (N = 131) by detection of IgM and IgG antibodies to *Borrelia* antigens by PHOSPHAN tests of sequential serum samples.

**Assay variant**	**Number (%) [95% CI] of patients with positive PHOSPHAN reactions by time after treatment** ^a^			**Number (%) [95% CI] of patients who seroconverted** ^b^
	**Baseline**	**≥8 days postbaseline**	**Total**	
*Bb* C6 IgM	32 (24.4) [17, 33]	11 (8.4) [4, 15]	43 (32.8) [25, 42]	22^c^ (51.2) [36, 67]
OspC IgM	24 (18.3) [12, 26]	28 (21.4) [15, 29]	52 (39.7) [31, 49]	39 (75.0) [61, 86]
VlsE IgM	16 (12.2) [7, 19]	29 (22.1) [15, 30]	45 (34.4) [26, 43]	41 (91.1) [79, 98]
*Bb* C6 IgG	51 (38.9) [31, 48]	44 (33.6) [26, 42]	95 (72.5) [64, 80]	84 (88.4) [80, 94]
*Bg* C6 IgG	39 (29.8) [22, 38]	38 (29.0) [21, 38]	77 (58.8) [50, 67]	65 (84.4) [74, 92]
*Ba* C6 IgG	50 (38.2) [30, 47]	45 (34.4) [26, 43]	95 (72.5) [64, 80]	85 (89.5) [82, 95]

*(Bb) B. burgdorferi, (Bg) B. garinii, (Ba) B. afzelii.*

^a^Statistically significant differences (Fisher's exact test, *p* < 0.05) were observed for comparison of pairs: *Bb* C6 IgM versus VlsE IgM, *Bb* C6 IgG, *Bg* C6 IgG or *Ba* C6 IgG for patients who seroconverted; OspC IgM versus *Bb* C6 IgG or *Ba* C6 IgG and VlsE IgM versus *Bb* C6 IgG, *Bg* C6 IgG and *Ba* C6 IgG for patients at the baseline; *Bb* C6 IgM, OspC IgM and VlsE IgM versus *Bb* C6 IgG, *Bg* C6 IgG and *Ba* C6 IgG for total number of patients.

^b^Seroconversion was ascertained by the appearance of IgM or IgG and/or no less than twofold increase or decrease in Lyme Index values in sequential serum samples.

^c^Number of patients who seroconverted out of the total number of patients with positive PHOSPHAN reactions.

Serum samples from 15 patients, collected at the onset of the disease prior to treatment, proved to contain C6-IgM alone. In nine of these patients, IgG appeared at a later stage of recovery, which was accompanied in some cases by seroconversion of IgM to OspC and VlsE. The period between tick bite and serum sampling varied from 15 to 21 days, averaging 17 days. No IgG was detected in the other six patients, but one of them showed a positive result in C6 ELISA, and seroconversion of IgM to VlsE and a stable level of IgM to OspC was observed in one patient each. Apparently, these patients failed to develop a complete immune response because of a shorter period from tick bite to the onset of the disease, compared to the first group (on average, 2 vs. 14 days), and, consequently, an earlier start of treatment with antibiotics, which is known to interfere with the production of specific antibodies [25, 26].

As inferred from *Bb* C6-IgG seroconversion, an active infection process was taking place in 84 out of 95 cases (88.4%). Seroconversion of IgM to *Bb* C6, OspC and VlsE proteins was confirmed in 51.2, 75 and 91% of patients, respectively (Table 3). Seroconversion ascertained by PHOSPHAN tests with two to six antigens simultaneously was observed in the majority of EM patients, thus increasing the reliability of serologic confirmation of LB.

## Discussion

We have described a new approach to simultaneous detection of the spectrum of specific IgM or IgG to *Borrelia* antigens using the technology of microarray phosphorescence analysis. The PHOSPHAN method measures the antibodies that bind to C6 peptides or recombinant proteins OspC and VlsE from three *Borrelia* genospecies (*B*. *burgdorferi*, *B*. *garinii*, and *B*. *afzelii*) printed as an array of tiny spots on the well bottoms of standard 96-well microplates. PHOSPHAN combines the advantages of ELISAs in sensitivity and of immunoblots in specificity. Similar to second-tier immunoblots, it allows the spectrum of anti-*Borrelia* antibodies to be detected simultaneously by analyzing the same serum sample; unlike immunoblots, it has no disadvantages connected with low sensitivity for stage I disease and subjective interpretation of weakly positive bands. Since the PHOSPHAN assay is similar to ELISA, it can be performed using standard laboratory equipment (washers, shakers, etc.) that makes it cost-effective. A broad dynamic range and high values of the Lyme Index (Fig 1) are advantages offered by PHOSPHAN (due to the use of luminescence detection and the high linear output of the Pt coproporphyrin reporter). This improves the “resolving” capacity of PHOSPHAN tests to distinguish between active and prior infection by detecting a significant difference in the Lyme Index values between sequential serum samples, with no need for labor-consuming end-point titration. Since the phosphorescent label remains stable for months, the results can be analyzed or reanalyzed long after the assay was completed. PHOSPHAN testing is a promising high-throughput screening tool for identifying antibodies to *Borrelia* antigens.

A single-tier immunoassay for the C6 peptide is currently regarded as a potential alternative to the conventional two-tier procedure of testing for Lyme disease [27, 28]. Therefore, our main purpose in this study was to evaluate the efficiency of the PHOSPHAN assay in detecting IgM and IgG to C6 in serum samples from EM patients. The results confirmed previous data [25, 29] that the immune response to this peptide is manifested mainly in production of IgG. The sensitivity and specificity of PHOSPHAN IgG detection with *B*. *burgdorferi* C6 were similar to those in C6 ELISA (Table 1 and Fig 2A). The proportion of positive samples with IgM, compared to IgG (M_1_ vs. G_1_) was significantly lower both at the baseline and at all times after the onset of the disease (Fig 2B). Detection of IgM in addition to IgG (M_1_G_1_) improved the sensitivity of PHOSPHAN assay to a level significantly exceeding that of C6 ELISA (Fig 2A) only at the earliest stage of disease prior to treatment (Fig 2C), with the specificity of both methods being approximately equal (Table 1). The detection of C6-IgM in the absence of C6-IgG is an extremely rare event. On the other hand, the appearance of C6-IgM in at least 9 out of 131 patients (6.9%) was preceded by production of C6-IgG, which has not been recorded in patients with Lyme disease caused by *B*. *burgdorferi* sensu stricto [25, 29].

The positive results of PHOSPHAN and C6 ELISA tests coincided for 180 out of 184 (97.8%) serum samples, and the negative results, for 122 out of 152 (88.4%) samples from LB patients. Divergent results were obtained for samples from 33 patients. In two cases, positive results in PHOSPHAN were recorded at a later stage than in C6 ELISA; in 25 cases, conversely, specific antibodies in C6 ELISA test were detected later, or a decrease in their titer below the limit of detection (*LI* < 0.9) was recorded earlier than in PHOSPHAN tests. In one patient, a positive result was obtained only in C6 ELISA; in another five patients, the results of C6 ELISA were negative, but PHOSPHAN confirmed seroconversion in these individuals.

These data provide evidence for higher sensitivity of the PHOSPHAN assay, which may be due to several factors. As follows from theoretical calculations [30], miniaturization into a microarray format improves the sensitivity of multiplex analysis, compared to conventional ELISAs, due to an increase in the local concentration of analytes in the dotlike reaction zone and proportional reduction of background signal from nonspecific components of the sample. The effect of these factors should be more noticeable when low concentrations of analytes are detected, as in the case of "early" antibodies (Fig 2C). Another relevant factor is that the C6 Lyme ELISA kit detects total IgM/IgG to C6, whereas these antibodies in PHOSPHAN tests are detected separately. Such differences may also have an effect on the sensitivity of the methods.

We observed no significant dependence of the sensitivity of IgG detection in PHOSPHAN assay on the *Borrelia* genospecies used as a source of C6. However, positive responses were recorded more frequently with C6 from *B*. *burgdorferi* or *B*. *afzelii* than from *B*. *garinii* despite minor differences in C6 sequence between the three genospecies [16] (Fig 4), as was also noted by other authors [15, 16]. In view of previous studies [15, 16, 31], it appears necessary to analyze a greater number of samples from different geographic regions and from patients with different forms of the disease to gain an insight into specific features of their interactions with C6 from the three *Borrelia* genospecies.

Data on the timing of production of IgM to recombinant OspC and VlsE proteins, compared to C6, are presented here for the first time. The contribution of C6-IgM to the total IgM immune response reached a peak at the baseline, and those of OspC-IgM and VlsE-IgM, during the second to fourth week after the start of antibiotic therapy (Fig 3B).

The frequency of antibody detection depended directly on the duration of incubation period and time from the onset of illness to first blood draw. As early as 1 week after tick bite, OspC-IgM, C6-IgM, and C6-IgG could already be detected in a few of the samples collected soon after the onset of the disease. An increase in the frequency of positive samples at later stages of recovery provided evidence for an active infection process [32]. Although OspC-IgM production a week after tick bite was demonstrated previously [33], such a short incubation period could be insufficient for the production of IgM and IgG to the C6 peptide [27]. Taking into account that these antibodies can persist for a long time in the absence of treatment [27, 34], early positive test results in some patients may be explained by previous contact with the pathogen.

Our study was limited to analysis of sera from EM patients in the acute period of the disease. It may well be that the multiplex phosphorescence assay will produce different results when analyzing samples from patients with the erythema-free form of LB, which occurs in Russia with a frequency of up to 45% [5], or samples taken from patients with later manifestations of the disease.

Thus, the multiplex phosphorescence immunoassay is a promising method for simultaneously revealing the spectrum of antibodies to several *Borrelia* antigens. A broad dynamic range and high values of the Lyme Index, speed and convenience are advantages offered by this method. The detection of IgM and IgG to the C6 peptide of *B*. *burgdorferi* provides effective serologic confirmation of LB and, with high probability, is indicative of an active infection process in EM patients. Additional PHOSPHAN tests for IgM to OspC and VlsE improve the information value and reliability of serologic analysis.

## Supporting Information

S1 Dataset The dataset underlying the findings reported in the manuscript. (XLS)

S1 Fig Schematic representation of PHOSPHAN tests design. Detection of IgM (A) or IgG (B) antibodies to *Borrelia* antigens printed on the well bottoms of standard 96 well microplates. (A) 1, IgM in patient sera; 2, monoclonal mouse antibodies to human IgM conjugated to biotin; 5, streptavidin conjugated to Pt coproporphyrin. (B) 3, IgG in patient sera; 4, monoclonal mouse antibodies to human IgG conjugated to biotin; 5, streptavidin conjugated to Pt coproporphyrin.
(TIF)

S2 Fig Exterior view of Diagem. This biochip analyzer was used in this study to register phosphorescence signals from the bottoms of standard 96 well microplates.
(TIF)

S3 Fig The patterns of phosphorescence signal distribution over the bottoms of standard 96 well microplate (detection of IgG antibodies to *Borrelia* antigens). An operator sees this picture on the screen of the computer attached to the biochip analyzer. Wells C12-D12 and E12-F12 are for positive control serum and negative control serum samples, respectively; the other wells are serum samples from EM patients. Each control or patient sample was examined in duplicate.
(TIF)
