# Supplementary figures and images for: C6 Peptide-Based Multiplex Phosphorescence Analysis (PHOSPHAN) for Serologic Confirmation of Lyme Borreliosis
Source: PLoS One. 2015 Jul 6;10(7):e0130048. doi: 10.1371/journal.pone.0130048 (PMC4492927; doi:10.1371/journal.pone.0130048)

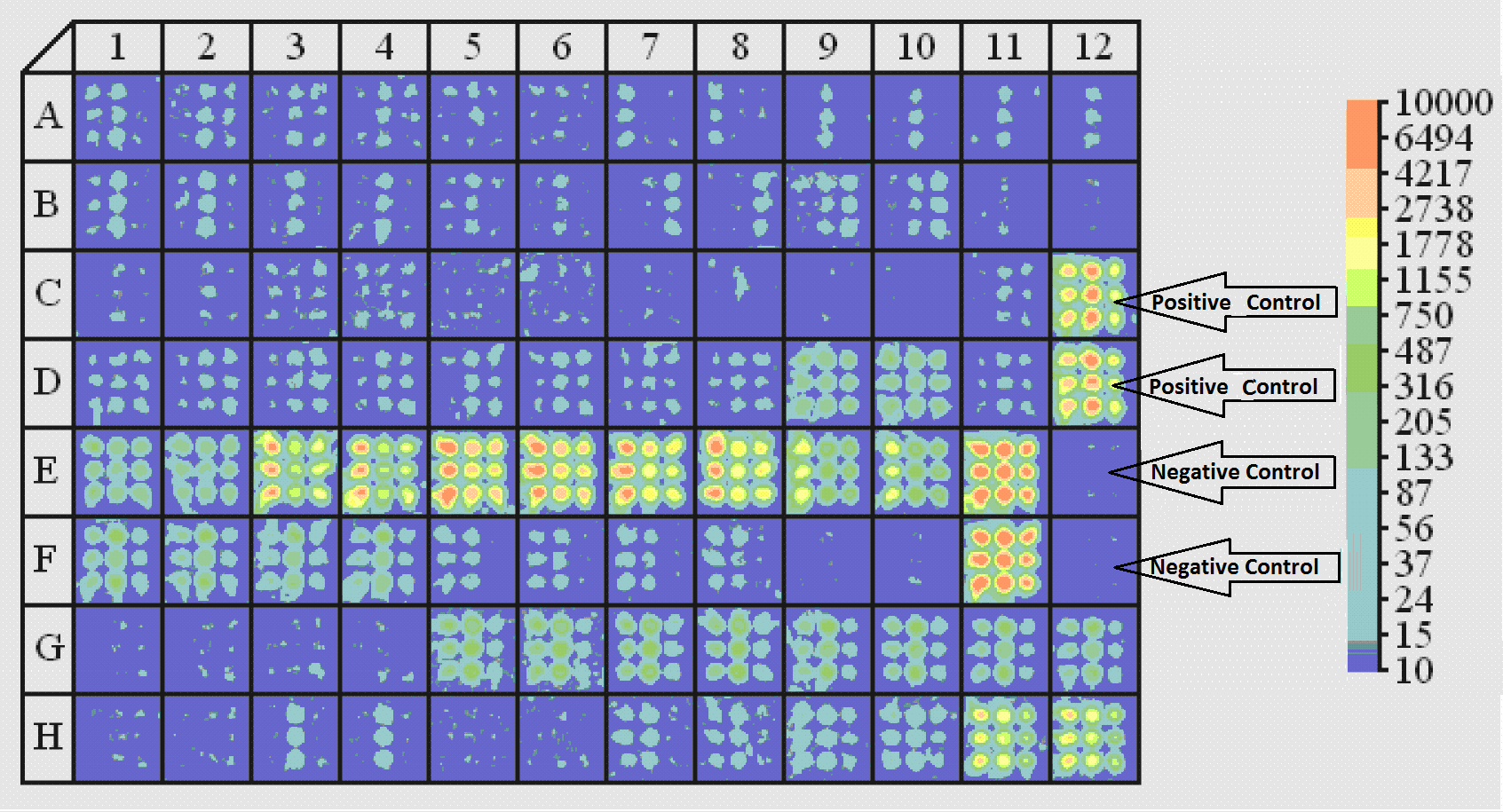

Supplement: S3 Fig — An operator sees this picture on the screen of the computer attached to the biochip analyzer. Wells C12-D12 and E12-F12 are for positive control serum and negative control serum samples, respectively; the other wells are serum samples from EM patients. Each control or patient sample was examined in duplicate. (TIF) [file pone.0130048.s004.tif]
